# Gut microbiota of liver transplantation recipients

**DOI:** 10.1038/s41598-017-03476-4

**Published:** 2017-06-19

**Authors:** Li-Ying Sun, Yun-Sheng Yang, Wei Qu, Zhi-Jun Zhu, Lin Wei, Zhi-Sheng Ye, Jian-Rui Zhang, Xiao-Ye Sun, Zhi-Gui Zeng

**Affiliations:** 1 Liver Transplantation Center, National Clinical Research Center for Digestive Diseases, Beijing Friendship Hospital, Capital Medical University, Beijing, China; 2 Institute of Digestive Diseases, Chinese PLA General Hospital, No. 28 Fuxing Road, Beijing, China; 3 Institute of Developmental Biology and Molecular Medicine, Fudan University, Shanghai, China; 4 Tianjin First Central Hospital, Tianjin, China

## Abstract

The characteristics of intestinal microbial communities may be affected by changes in the pathophysiology of patients with end-stage liver disease. Here, we focused on the characteristics of intestinal fecal microbial communities in post-liver transplantation (LT) patients in comparison with those in the same individuals pre-LT and in healthy individuals. The fecal microbial communities were analyzed via MiSeq-PE250 sequencing of the V4 region of 16S ribosomal RNA and were then compared between groups. We found that the gut microbiota of patients with severe liver disease who were awaiting LT was significantly different from that of healthy controls, as represented by the first principal component (p = 0.0066). Additionally, the second principal component represented a significant difference in the gut microbiota of patients between pre-LT and post-LT surgery (p = 0.03125). After LT, there was a significant decrease in the abundance of certain microbial species, such as *Actinobacillus*, *Escherichia*, and *Shigella*, and a significant increase in the abundance of other microbial species, such as Micromonosporaceae, Desulfobacterales, the *Sarcina* genus of Eubacteriaceae, and *Akkermansia*. Based on KEGG profiles, 15 functional modules were enriched and 21 functional modules were less represented in the post-LT samples compared with the pre-LT samples. Our study demonstrates that fecal microbial communities were significantly altered by LT.

## Introduction

With the development of sequencing techniques, accumulating research on the connection between the human gut microbiota and disease has been conducted. This research includes studies on the association of gut microbiota alterations with liver diseases, such as obesity-related liver disease^1^, non-alcoholic fatty liver disease^2, 3^, autoimmune liver disease^4^, and liver cirrhosis^5, 6^. Liver cirrhosis is characterized by the pathological changes of end-stage liver diseases, and its clinical manifestations include a series of complications associated with portal hypertension, such as hepatic encephalopathy, spontaneous bacterial peritonitis, portal hypertensive gastropathy, and bleeding from esophageal varices. Many studies have reported that these clinical manifestations are associated with the intestinal microbial community^7–10^. Liver transplantation (LT) resolves the associated problems in patients with liver failure or portal hypertension; following LT, the liver function of patients gradually returns to normal and portal hypertension-related gastrointestinal diseases can be alleviated^11^.

Currently, no study has reported on the differences in the intestinal microbiota of patients who received LT, as measured using 16S ribosomal RNA (rRNA) sequencing technology. Lu *et al*.^12^ analyzed the variation in the microbiome during the perioperative period in LT patients by performing denaturing gradient gel electrophoresis (DGGE), and their results showed that the diversity in predominant intestinal microbes decreased substantially in patients who experienced infection during the perioperative period.

Using MiSeq-PE250 sequencing technology combined with bioinformatics analysis, we studied the characteristics of the intestinal microbiota from patients with end-stage liver disease before and after they received LT. The purpose of this study was to analyze the differences in the intestinal microflora of patients between pre-LT and post-LT to investigate the effect of LT therapy and subsequent immunosuppressive treatment on the intestinal microflora, thereby providing experimental data further revealing the mechanism by which liver diseases alter the gut microbiota.

## Methods

### Study population

This study included 33 samples from 24 participants, nine of whom initially had an end-stage liver disease (1 sample each pre- and post-LT, for a total of 18 samples) and 15 of whom were healthy controls. All details of the entire study design and procedures involved were established in accordance with the Declaration of Helsinki. Written informed consent forms were signed before the time of sample collection. This protocols were approved by the Ethics Committee of Tianjin First Central Hospital. Of the nine LT patients, four had liver failure because of decompensated cirrhosis, and five had hepatocellular carcinoma (HCC) (the characteristics of the patients are presented in Supplementary Tables 1, 2 and 3). All patients were undergoing primary non-bypass orthotopic LT for the first time, and the method of biliary anastomosis was end-to-end anastomosis. Furthermore, all nine patients were male and 49.4 ± 13.9 years old. Before LT, the patients provided fecal samples and underwent blood tests. Each patient was re-examined 3 months after LT, at which time the patients provided a second fecal sample. The 15 healthy controls were individuals who visited the physical examination center of the hospital during the study period and who had normal blood test results. Among the 15 healthy controls, 11 were male and 4 were female, aged 48 ± 4.19 years (see Supplementary Table 4). There was no significant difference (see Supplementary Tables 5 and 6). None of the individuals in this study used antimicrobial agents in the month prior to enrollment or had any evidence of systemic bacterial, viral, or fungal infection. All post-LT patients received immunosuppression therapy with tacrolimus and methylprednisolone. In the first 5 to 7 days after surgery, the patients received antibiotic treatment with cephalosporin to prevent infection. No vascular complications or infections occurred through the time of patient discharge.

### Sampling and DNA extraction

Fecal samples were frozen at −80 °C immediately upon collection and then stored for later use. At the beginning of the experiment, 180–200 mg of each sample was weighed out and transferred to a 2-ml centrifuge tube, which was then placed on ice. DNA was extracted from the samples using the repeated bead beating plus column (RBB+C) method. Briefly, ASL buffer (1.4 ml) was added to each sample, followed by the addition of 0.4 g of sterile zirconia beads (0.3 g of 0.1-mm beads and 0.1 g of 0.5-mm beads). The samples were subjected to bead beating (3 min, maximum speed) using a Mini-BeadBeater-16 cell disruptor (BioSpec Products, Bartlesville, OK, USA) prior to an initial incubation for lysis via heating at 70 °C for 15 min, with thorough shaking every 5 min. Subsequent steps of the DNA extraction protocol followed the instructions from the manufacturer of the QIAamp Stool Mini Kit (Qiagen, Germany) for bacterial DNA extraction.

### 16S rRNA gene amplicon sequencing using a MiSeq sequencer

For sequencing, isolated fecal DNA was used as a template for amplification, and the V4 region of 16S rRNA was amplified by performing PCR assays using the universal bacterial primer set 515F (5′-GTGCCAGCMGCCGCGGTAA-3′) and 806R (5′-GGACTACHVGGGTWTCTAAT-3′).

The PCR reaction mixture (30 μl) contained 2× High-Fidelity PCR Master Mix, 3 µl of primer (2 μM), and 10 μl of gDNA, and the reaction steps were as follows: 98 °C for 1 min, followed by 35 cycles of 98 °C for 10 s, 50 °C for 30 s, and 72 °C for 30 s, with a final elongation at 72 °C for 5 min. The reactions were conducted in a Veriti 96-Well Thermal Cycler (Biometra, Germany). The resulting PCR products were purified via separation on a 2% agarose gel, followed by DNA isolation using the QIAquick Gel Extraction Kit (Qiagen, Santa Clarita, CA, USA) according to the manufacturer’s instructions. The purified DNA was quantified using a Qubit® 2.0 Quantitation Starter Kit (Invitrogen, USA) according to the manufacturer’s instructions. The partial 16S rRNA genes were sequenced using an Illumina MiSeq sequencer.

### Bioinformatics analysis

#### Data processing

Paired end reads were overlapped using FLASH-1.2.10^13^ with the parameters ‘-m 150 -M 250 -x 0.15 -O’; no mismatches in primers or barcodes were allowed, and no ambiguous bases (N) in whole overlapped reads were allowed. When assigning reads to samples, the barcodes had to be consistent with the reference barcode sequences. Finally, we removed chimeras using Uchime^14^.

#### Binning of operational taxonomic units (OTUs)

Usearch^15^ was used to bin reads into OTUs with a 0.97 identity cut-off. Samples with more sequenced reads had more observed OTUs (rho = 0.78; *p* = 2.533 × 10^−07^, Spearman’s rank correlation analysis) when all reads were binned into OTUs. Thus, we randomly chose reads with the same number (10,000) and then identified representative OTU reads. Finally, we mapped all randomly chosen reads against representative OTU reads to obtain the OTU composition of all samples.

#### Diversity and richness calculation

Shannon, simpson, and invsimpson indices were calculated using the ‘vegan’ package in R^16^ with a normalized OTU matrix. The observed species, Chao1, and ICE indices were calculated using the ‘fossil’ package in R^17^ with a non-normalized OTU matrix.

#### Beta-diversity calculation

The Hellinger distance was calculated with the ‘topicmodels’ package in R^18^. The JSD distance was calculated with a custom R script provided by the European Molecular Biology Laboratory enterotyping tutorial (http://enterotype.embl.de/enterotypes.html). Spearman’s rank correlation distance was calculated using the following command in R: ‘D < −1 - cor.test (X, method = “spearman”)’, where X is the OTU composition of all samples. All three distances were calculated based on the OTU composition.

To calculate the unifrac distance, we constructed an abundance profile of unique reads from all randomly chosen reads (singletons were removed). We used MUSCLE v3.8.31^19^ to generate multiple alignments with the default parameters and used FastTree version 2.1.3 SSE3^20^ to construct a phylogenetic tree with a generalized time-reversible model. Finally, we used the ‘phyloseq’ package in R^21^ to calculate the unifrac distance with the following command: ‘Weighted_Unifrac_distance < -UniFrac (x1, weighted = T, normalized = T, fast = T)’, where ‘x1’ is a combination of the phylogenetic tree and the abundance of unique reads (the ‘weighted = F’ parameter was used to calculate the unweighted unifrac distance).

#### KEGG orthology and pathway profiles

KEGG orthology profiles were constructed using the piCrust version 1.0.0 pipeline^22^. First, our custom representative OTU reads were aligned against the greenGene_Database_16S_rRNA Fasta reference database (2013 version), and the abundances of representative OTU reads from the same 16S rRNA reference were then summed. The reference profile normalized by the 16S rRNA copy number was used to predict the KEGG orthology profile, and HUMAnN software version 0.99^23^ was subsequently used to determine the abundance of KEGG pathways and modules.

#### Statistical analysis

The Wilcoxon rank sum test based on the null hypothesis was performed to evaluate alpha diversity, principal coordinates, and KEGG module profiles between the different cohorts. A paired test was used to compare samples from the same individual (pre-LT and post-LT cohorts) with the following command in R: ‘wilcox.test (a, b, paired = T, alternative = “less”)’, where a and b are numeric vectors of the data for the pre-LT and post-LT samples, respectively. LEfSe^24^ was used to compare the bacterial populations between different cohorts. When using LEfSe to perform a Wilcoxon rank sum test between the pre-LT and post-LT samples, we made a replace correction using the *p*-values generated from a paired test.

#### Enterotype clustering

We used the ‘NbClust’ package in R^25^ to identify the number of clusters into enterotypes based on the OTU composition. We then used an exhaustive combination of two clustering methods (k-means method and complete cluster linkage method) and five different beta-diversities (weighted unifrac distance, unweighted unfired distance, JSD distance, Spearman’s rank correlation distance, and Euclidean distance), with indices integrated in NbClust to confirm the optimal number of clusters. Finally, the number of clusters was identified according to a major rule.

## Results

### DNA sequencing results

All 33 samples were pooled into one sequencing library, and a total of 1,907,569 raw reads were sequenced. After quality control and chimera removal, 1,281,639 reads remained for downstream analysis (Supplementary Table 7). To avoid bias caused by sequencing unevenness, we randomly downsized the sequences of all samples at the same level with a minimum cut-off number of 10,000.

Randomly chosen reads were binned into OTUs with a 0.97 identity cut-off, and 505 OTUs were considered to be representative OTUs. We then mapped all clean reads (1,281,639 reads before downsizing) against these representative reads, and 90.341% of reads were able to be re-binned. No significant differences in microbial richness (observed species, Chao1, and ICE indices) or diversity (shannon, simpson, and invsimpson indices) were found between any of the three cohorts (pre-LT patients, post-LT patients, and healthy controls) (*p* < 0.05) (Table 1).

**Table 1:** PCoA based on OTU composition.

P value	pre_control	pre_post	control_post
PC1	0.00661413	0.375	0.36840933
PC2	0.05555165	0.03125	0.07771601
PC3	0.83659349	0.29688	0.26659396

### Changes to gut microbiota composition in recipients of LT surgery

**Figure 1:**
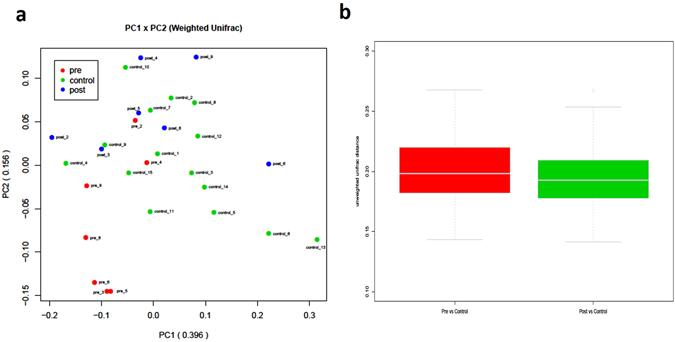
The results of a PCoA based on OTU composition are shown as graphs of the (**a**) LT-filtered weighted unifrac distance and (**b**) the unifrac distance from the pre-LT and post-LT samples to the control samples.

Samples pre_7 and post_1 were considered to be outliers and were excluded from our analysis. We also excluded the corresponding samples from the same two individuals: post_7 and pre_1 (for details, see Supplementary Figs 1 and 2). After exclusion of these samples, 29 (7 pre-LT, 7 post-LT, and 15 control samples) remained for downstream analysis. The results of a principal component analysis (PCoA; weighted unifrac distance) based on OTU composition showed that the gut microbiota of patients with severe liver disease who were awaiting LT surgery (pre-LT) were significantly different from that of control patients, which corresponded to the first principal component (PC1, *p* = 0.0066). Additionally, a second principal component (PC2) corresponded to a significant difference in the gut microbiota of the same patients between pre-LT and post-LT (*p* = 0.03125). However, there was no significant difference in the top three principal components between the post-LT patients and the controls (Fig. 1a and Table 1). We observed a reduction in the unweighted unifrac distance between the post-LT and control groups compared with that between the pre-LT and control groups (Fig. 1b, *p* = 0.03823), indicating that the gut microbiota composition of LT recipients became more similar to that of healthy controls after the LT surgery.

**Figure 2:**
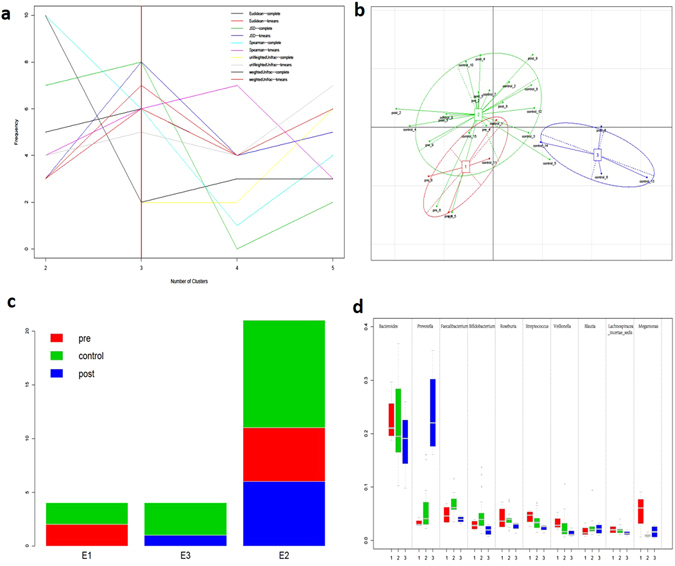
The enterotypes of samples from the pre-LT, post-LT, and healthy control groups were analyzed. (**a**) The number of enterotype clusters. (**b**) The enterotypes of all samples. (**c**) The distribution of enterotypes. (**d**) The genus distribution of all enterotypes.

Previous studies have demonstrated a clear stratification of human gut microbiota compositions, i.e., enterotypes, in populations that is neither nation- nor continent-specific^26^. Thus, we aimed to determine whether enterotype is associated with the changes in the gut microbiota observed in a patient after LT surgery. All samples could be clustered into three enterotypes according to a major rule (Fig. 2a and b, weighted unifrac distance, k-means clustering method). We found that the enterotype distribution was not associated with LT surgery (Fig. 2c, Fisher’s exact test of the pre-LT and post-LT groups). Based on the relative abundance of *Bacteroides*, *Prevotella*, and *Faecalibacterium* in the gut microbial population, we clustered the samples into three enterotypes (Fig. 2d). While the enterotype of three recipients (cases 3, 8, and 6) changed following LT, the gut microbiotas of two of these individuals (3 and 8) had completely recovered (shifted from E1 to E2 with significantly reduced beta-diversity). For case 6, although the weighted unifrac distance from the healthy control group was not significantly changed, the sample after LT surgery (post_6) was binned into E3, which otherwise consisted solely of healthy controls.

Of the four recipients without an enterotype change after receiving LT (cases 2, 4, 5, and 9), the gut microbiota of two cases (2 and 4) significantly skewed from that of the healthy controls after receiving LT surgery (beta-diversity was significantly increased). Furthermore, these two samples (pre_2 and pre_4) were homogeneously mixed with the control samples (Fig. 1a), indicating that LT surgery might have slightly disturbed the gut microbiota in these two cases. A follow-up trial showed that biliary complications (biliary anastomotic stricture) occurred in these two samples, but our data are insufficient to confirm an association between the gut microbiota and biliary complications. The gut microbiota of case 5 (post_5) had also recovered, even though no enterotype change was observed (Table 2).

**Table 2:** Changes to gut microbiota composition following LT surgery.

Case	Enterotype change^a^	P value^b^	More similar
2	E2-E2	1.72E-05	Pre^c^
4	E2-E2	0.033748	Pre
3	E1-E2	0.011748	Post^d^
5	E2-E2	4.49E-05	Post
8	E1-E2	0.000132	Post
6	E2-E3	0.564903	None^e^
9	E2-E2	0.10084	None

^a^Enterotype change represents the enterotype of individual samples taken before (pre, left) and after (post, right) LT.

^b^Based on a unilateral Wilcoxon rank sum test of the weighted unifrac distances between the pre-LT or post-LT sample and samples from the 15 healthy controls.

^c^Pre indicates that the weighted unifrac distance from the pre-LT sample to the healthy control samples is shorter than that from the post-LT sample to the healthy controls samples.

^d^Post indicates that the weighted unifrac distance from the post-LT sample to the healthy control samples is shorter than that from the pre-LT sample to the healthy control samples.

^e^None indicates that there is no significant difference (*p* > 0.05) in the weighted unifrac distances to the samples from the 15 healthy controls between the pre-LT and post-LT samples.

### Differential species abundance

We used a LEfSe analysis to detect differential abundances of species between the pre-LT, post-LT, and control samples. Because the pre-LT and post-LT samples belonged to the same individuals, we performed a correction for unilateral inspection, using the results of a Wilcoxon rank sum test in our LEfSe analysis. We then identified differential distributions of species using the default cut-offs (LDA score > 2.0; *p* value < 0.05).

**Figure 3:**
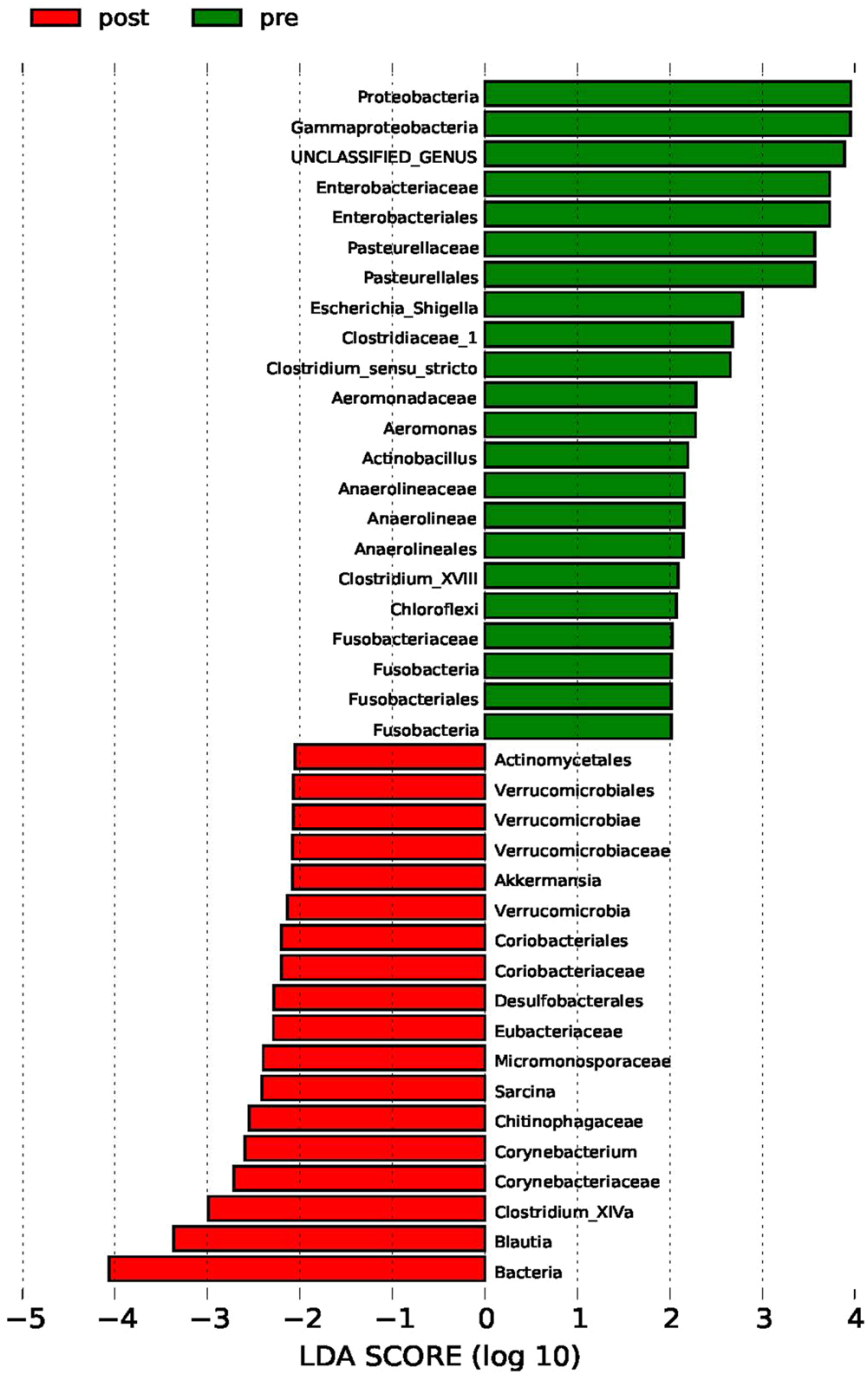
LEfSe analysis of differential species abundance between the pre-LT and post-LT groups was performed. The LDA score is used as the abscissa, and higher scores indicate a greater difference. The red and green horizontal bars represent the species enrichment in the post-LT and pre-LT groups, respectively.

**Figure 4:**
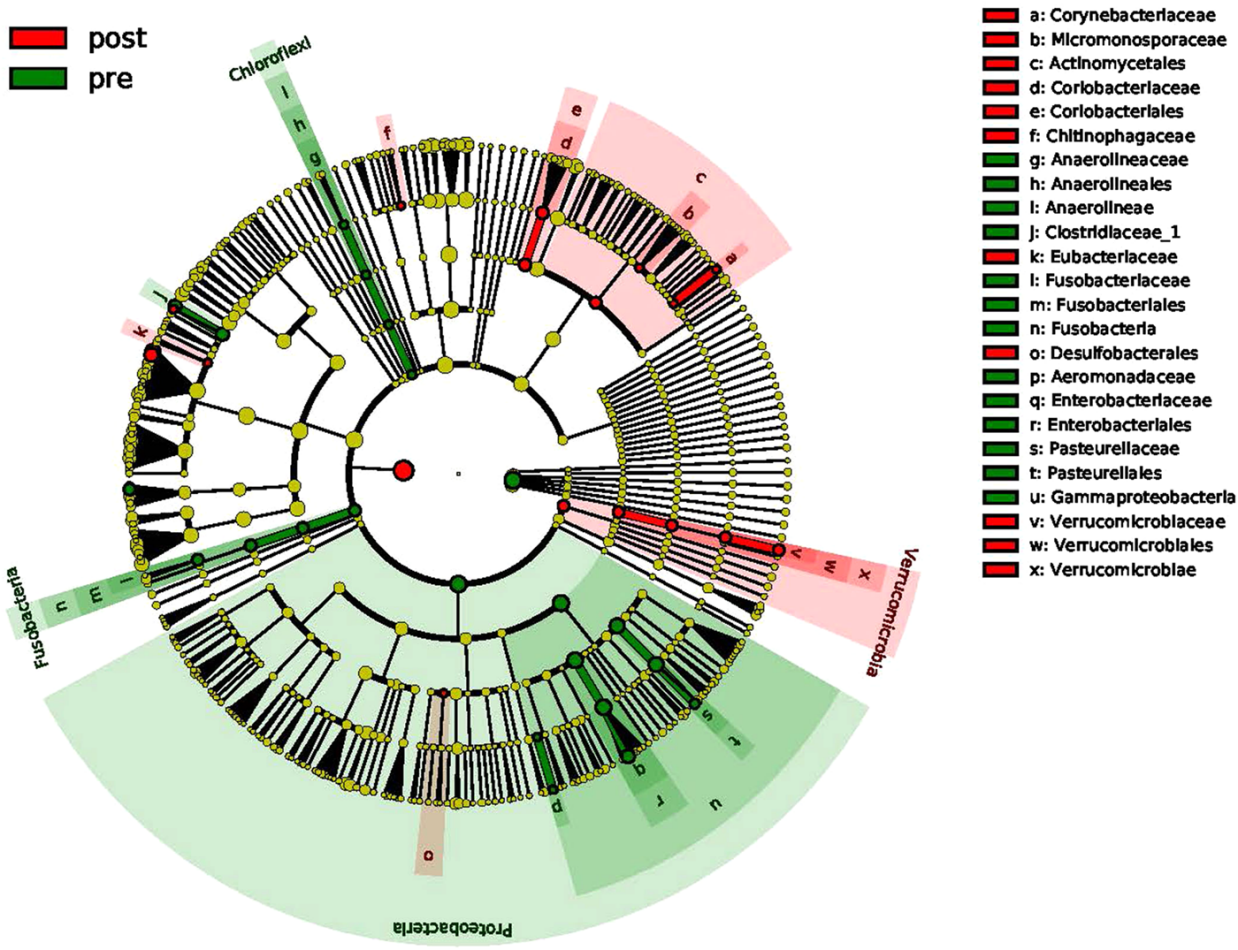
A cladogram plot of differential species abundance between the pre-LT and post-LT groups is shown. The red and green areas represent the species enrichment in the post-LT and pre-LT groups, respectively.

**Figure 5:**
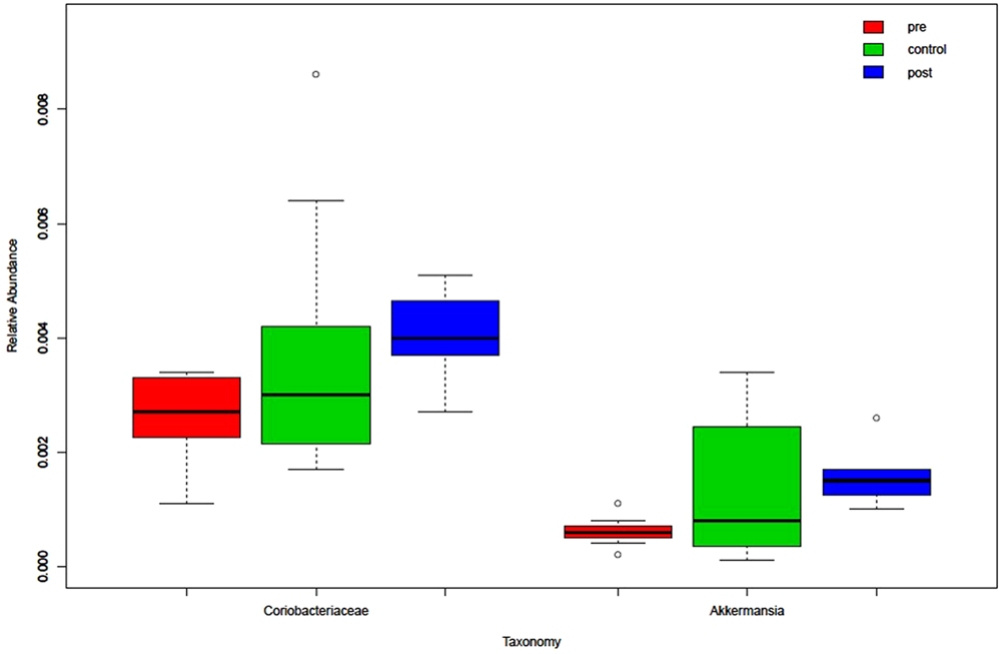
The distributions of Coriobacteriaceae and *Akkermansia* in all samples from the pre-LT, post-LT, and healthy control groups are shown.

**Figure 6:**
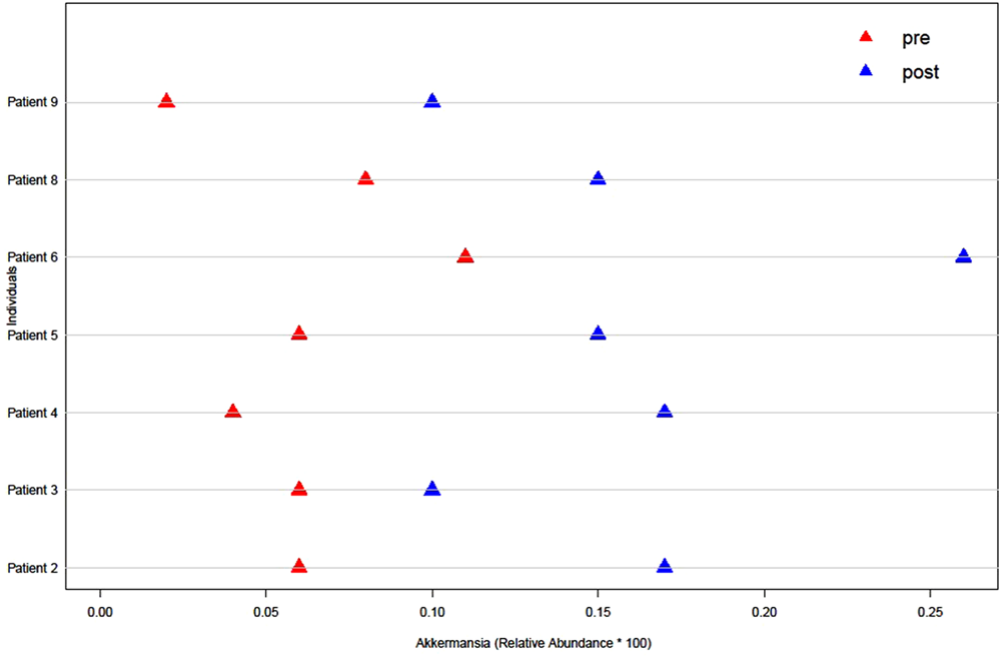
The abundance of *Akkermansia* in samples from patients collected before and after LT surgery is shown.

We found that the relative abundances of *Actinobacillus*, *Escherichia*, *Shigella*, Anaerolineaceae, Fusobacteriales, *Clostridium* (*sensu stricto*), Fusobacteriaceae, *Aeromonas*, and *Clostridium* cluster XVIII, among others, were significantly decreased but that the relative abundances of Micromonosporaceae, Desulfobacterales, Eubacteriaceae, *Sarcina*, *Akkermansia*, Chitinophagaceae, and Coriobacteriaceae were significantly increased in post-LT samples compared with pre-LT samples (Figs 3 and 4). Notably, there were no differences in the relative abundances of these species between the post-LT samples and the control samples (Fig. 5; low-abundance species are not shown). Although not all of the cases showed an enterotype change following the LT surgery, the relative abundance of *Akkermansia* changed after LT surgery in all individuals (mean fold-change: 2.92, range: 1.67–20.49; Fig. 6). Additionally, the relative abundances of *Clostridium* cluster XIVa, *Corynebacterium*, and *Blautia* were significantly increased following LT surgery, and their relative abundances in the post-LT samples were greater than those in the control samples.

### Differences in metabolic pathways between groups

We transformed the composition of the OTU sequence into KEGG orthology to analyze the differences in the metabolic pathways represented in the gut microbiota between pre-LT and post-LT samples. We generated the KEGG profiles and then compared the components of functional genomics in KEGG pathways, yielding 118 KEGG modules. Of these, 15 functional modules were enriched in the post-LT samples and 21 functional modules were less represented in the post-LT samples compared with the pre-LT samples (Tables 3 and 4).

**Table 3:** Modules enriched in post-LT samples compared with pre-LT samples.

Enriched post-LT	*P*	Functional description
M00233	0.0234375	Glutamate_transport_system
M00051	0.0234375	Uridine_monophosphate_biosynthesis,_glutamine_(+_PRPP)_=>_UMP
M00144	0.0234375	Complex_I_(NADH_dehydrogenase),_NADH_dehydrogenase_I
M00232	0.0234375	General_L-amino_acid_transport_system
M00088	0.0234375	Ketone_body_biosynthesis,_acetyl-CoA_=>_acetoacetate/3-hydroxybutyrate/acetone
M00342	0.029529115	Bacterial_proteasome
M00176	0.029529115	Sulfur_reduction,_sulfate_=>_H2S
M00116	0.029586034	Menaquinone_biosynthesis,_chorismate_=>_menaquinone
M00206	0.029586034	Cellobiose_transport_system
M00036	0.029586034	Leucine_degradation,_leucine_=>_acetoacetate_+_acetyl-CoA
M00200	0.029586034	Sorbitol/mannitol_transport_system
M00156	0.029586034	Complex_IV_(Cytochrome_c_oxidase),_cytochrome_c_oxidase,_cbb3-type
M00174	0.029586034	Methane_oxidation,_methylotroph,_methane_=>_CO2
M00277	0.0390625	PTS_system,_N-acetylgalactosamine-specific_II_component
M00033	0.046746242	Ectoine_biosynthesis

**Table 4:** Modules enriched in pre-LT samples compared with post-LT samples.

Enriched pre-LT	*P*	Functional description
M00017	0.0078125	Methionine_biosynthesis,_aspartate_=>_homoserine_=>_methionine
M00229	0.0078125	Arginine_transport_system
M00019	0.0078125	Leucine_biosynthesis,_pyruvate_=>_2-oxoisovalerate_=>_leucine
M00324	0.0078125	Dipeptide_transport_system
M00150	0.0078125	Complex_II_(succinate_dehydrogenase_/_fumarate_reductase),_fumarate_reductase
M00121	0.0078125	Heme_biosynthesis,_glutamate_=>_protoheme/siroheme
M00064	0.0078125	ADP-L-glycero-D-manno-heptose_biosynthesis
M00025	0.0156250	Tyrosine_biosynthesis,_chorismate_=>_tyrosine
M00260	0.0234375	DNA_polymerase_III_complex,_bacteria
M00335	0.0234375	Sec_(secretion)_system
M00136	0.0234375	GABA_biosynthesis,_prokaryotes,_putrescine_=>_GABA
M00230	0.0234375	Glutamate/aspartate_transport_system
M00060	0.0234375	Lipopolysaccharide_biosynthesis,_KDO2-lipid_A
M00276	0.0390625	PTS_system,_mannose-specific_II_component
M00336	0.0390625	Twin-arginine_translocation_(Tat)_system
M00008	0.0390625	Entner-Doudoroff_pathway,_glucose-6P_=>_glyceraldehyde-3P_+_pyruvate
M00117	0.0390625	Ubiquinone_biosynthesis,_prokaryotes,_chorismate_=>_ubiquinone
M00226	0.0390625	Histidine_transport_system
M00225	0.0390625	Lysine/arginine/ornithine_transport_system
M00300	0.0390625	Putrescine_transport_system
M00012	0.0390625	Glyoxylate_cycle

## Discussion

The microbes in our bodies are estimated to collectively account for up to 100 trillion cells and may encode 100-fold more unique genes than our own genome^27–29^. Not surprisingly, the intestinal microflora play key roles in the maintenance of a healthy state, including influencing the absorption of food and the synthesis and digestion of polysaccharides, vitamins, and other composite materials^30–32^. The human intestine and liver are closely related and interact with each other through bile, hormones, inflammatory mediators, and products of digestion and absorption. Notably, the amount, type, and composition of intestinal microflora can have both direct and indirect effects on the physiological function of the liver and can impact the occurrence and development of liver diseases^2, 4, 33^.

In patients with end-stage liver disease, liver cells show degeneration, necrosis, and other pathological changes that damage the normal liver structure, ultimately leading to cirrhosis and portal hypertension. Increased resistance to portal blood flow is the primary factor in the pathophysiology of portal hypertension, and it mainly develops via morphological changes that occur in chronic liver diseases. Another factor contributing to the exacerbation of portal hypertension is significantly increased portal blood flow, which is caused by arteriolar splanchnic vasodilation and hyperkinetic circulation^34^. Due to portal hypertension, portal hypertensive gastropathy and bowel disease can appear. In addition, intestinal mucosal damage causes an increase in mucosal permeability and a decrease in defense functions, resulting in bacterial translocation and changes to substance metabolism^35^.

Grąt *et al*. investigated LT candidates and revealed that pre-LT dysbiosis was significantly correlated with *Enterococcus* ^36^. Several other studies have also revealed that Enterobacteriaceae and *Enterococcus* were more abundant in LT candidates with liver cirrhosis than in healthy individuals. Furthermore, potentially pathogenic Enterobacteriaceae have been described as major determinants of gut dysbiosis in cirrhosis patients and have been identified as harmful components of the intestinal microbiota^37–39^. However, current knowledge on the gut microbiota and its alterations in relation to post-LT outcomes including early infections, acute cellular rejection and long-term complications are insufficient due to the limited number of human trials and small sample sizes^40^.

The results of our PCoA based on OTU composition showed that the gut microbiota of patients with severe liver disease who were awaiting LT surgery was significantly different from that of control subjects; however, there was no significant difference in the gut microbiota between post-LT and control samples. We also found significantly decreased relative abundances of certain species following LT, including *Actinobacillus*, *Escherichia*, *Shigella* (belonging to Enterobacteriaceae), and Fusobacteriales. However, the relative abundances of other species, such as *Akkermansia* and *Corynebacterium*, were significantly increased after LT. Notably, the abundance of *Akkermansia* was higher in all post-LT samples.

Several recently published reports in human and animal settings indicated that the abundance of *Escherichia* species may be related to the presence of HCC^41^. Our study comparing HCC and non-HCC samples showed no significant differences in the overall OTU composition of the gut microbiota between HCC and non-HCC samples. In particular, *Escherichia* is not enriched in HCC samples, and this finding is not consistent with some other studies. We propose that cirrhosis might be the major symptom of HCC patients with liver cirrhosis. A previous study showed that *Escherichia* is enriched in the gut microbiota of liver cirrhosis patients, compared with healthy controls; thus, we are unsure whether *Escherichia* is solely associated with liver carcinoma. However, the abundance of *Escherichia* was significantly decreased after LT surgery (the fold-change in abundance was not significantly different between HCC and non-HCC samples), and this decrease might be partially attributable to LT in patients with liver cirrhosis prior to the LT surgery. Future studies will need an expanded sample size, as the sample size of the current study was small.

*Akkermansia* plays a role in intestinal protection; it can consolidate epithelial cells to strengthen the intestinal barrier. This species has the capacity not only to degrade mucins but also to stimulate mucin synthesis, which is illustrative of an autocatalytic process^42^. *Akkermansia* spp. appear to be important for a healthy mucus layer in the human gut with respect to mucus production and thickness. The mucus layer is continuously reshaped and refreshed, and in this way, a healthy environment for the underlying epithelial cells is created^43^.

Some *Corynebacterium* spp., such as *Corynebacterium glutamicum*, which can utilize a variety of carbohydrates and organic acids as its sole source of carbon and energy, can also be of benefit to their hosts^44^. *Corynebacterium glutamicum* can produce amino acids including L-glutamic acid, L-threonine, and L-lysine^45^. L-glutamic acid is involved in protein and sugar metabolism, promotes oxidation processes, and can combine with ammonia in the body to form non-toxic L-glutamine, thereby decreasing blood ammonia levels.

We generated KEGG profiles and compared the representations of KEGG functional pathways. In the post-LT samples, 15 functional modules were enriched and 21 function modules were less represented compared with the pre-LT samples. For example, M00116 is a member of a metabolic pathway for the synthesis of vitamin K, which is a major cofactor that can help convert prothrombin into thrombin through a series of enzymatic reactions. Importantly, intestinal bacterial compounds are a main source of material for vitamin K production. Previous reports indicated that the synthesis of vitamin K increases and blood clotting function improves in patients after LT^46, 47^. M00232/M00233 is an amino acid transporter in a metabolic system. The increased amino acid metabolism in patients after LT indicates that the level of intestinal nutrients is increased and the digestive and absorptive functions of the intestinal tract are improved in these patients^48, 49^.

Liver function gradually improves and portal venous pressure is relieved in recipients after LT. Our study indicates that fecal microbial communities were significantly altered following LT and that the gut microbiota composition of LT recipients was more similar to that of healthy controls after the LT surgery. The current study is limited by its small sample size, which prevents subgroup analysis. Therefore, further research should be conducted.

## Electronic supplementary material

Supplementary information
