# Supplementary material for: Gut microbiota of liver transplantation recipients
Source: Sci Rep. 2017 Jun 19;7:3762. doi: 10.1038/s41598-017-03476-4 (PMC5476624; doi:10.1038/s41598-017-03476-4)
Supplement: Supplementary file 1 — Supplementary information [file 41598_2017_3476_MOESM1_ESM.pdf]

Gut microbiota of liver transplantation recipients

Li-Ying Sun[1], Yun-Sheng Yang[2*], Wei Qu[1], Zhi-Jun Zhu[1], Lin Wei[1], Zhi-sheng Ye[3], Jian-rui Zhang[1], Xiao-ye Sun[4], Zhi-Gui Zeng[1]

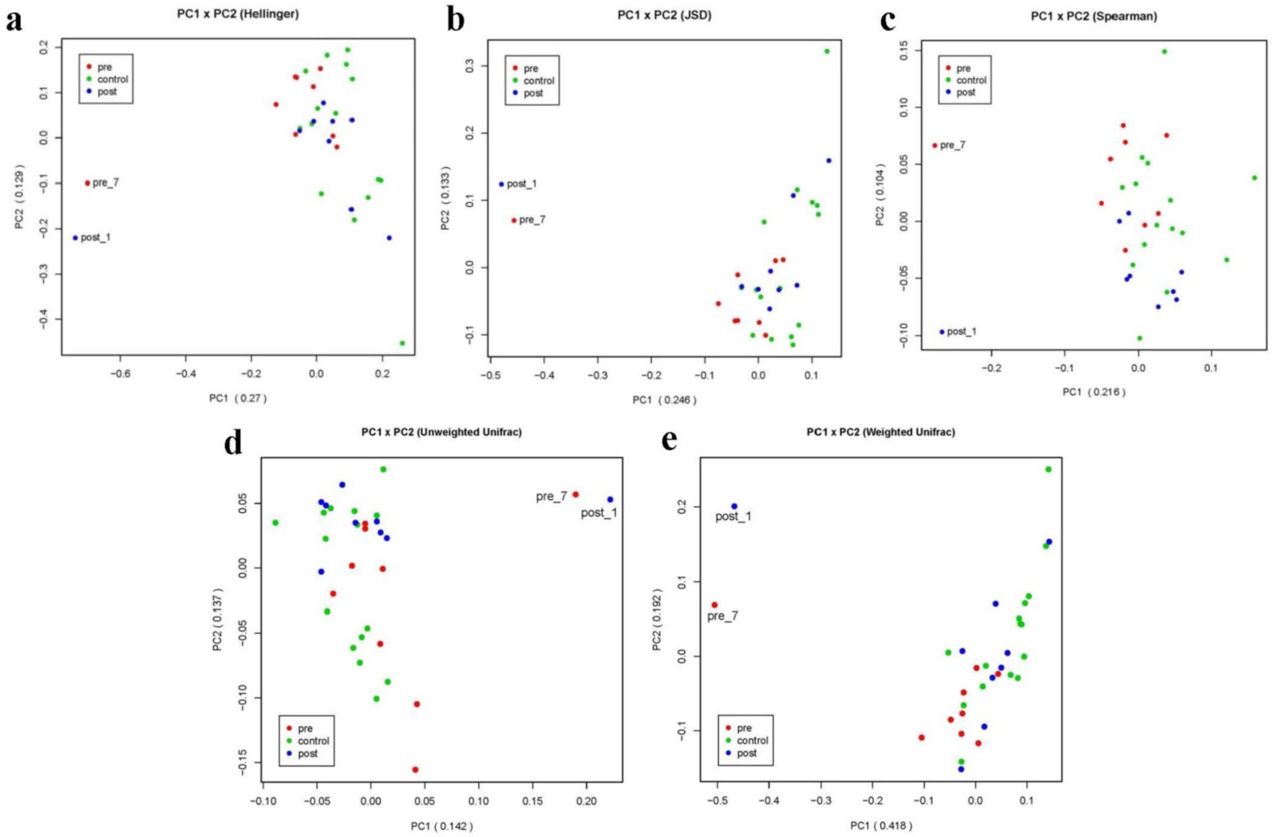

Supplementary Fig.1 a) PCoA analysis based on Hellinger distance. b) PCoA analysis based on JSD distance. c) PCoA analysis based on Spearman Coefficient Distance. d) PCoA Based on Unweighted Unifrac distance. e) PCoA analysis based on weighted unifrac analysis.

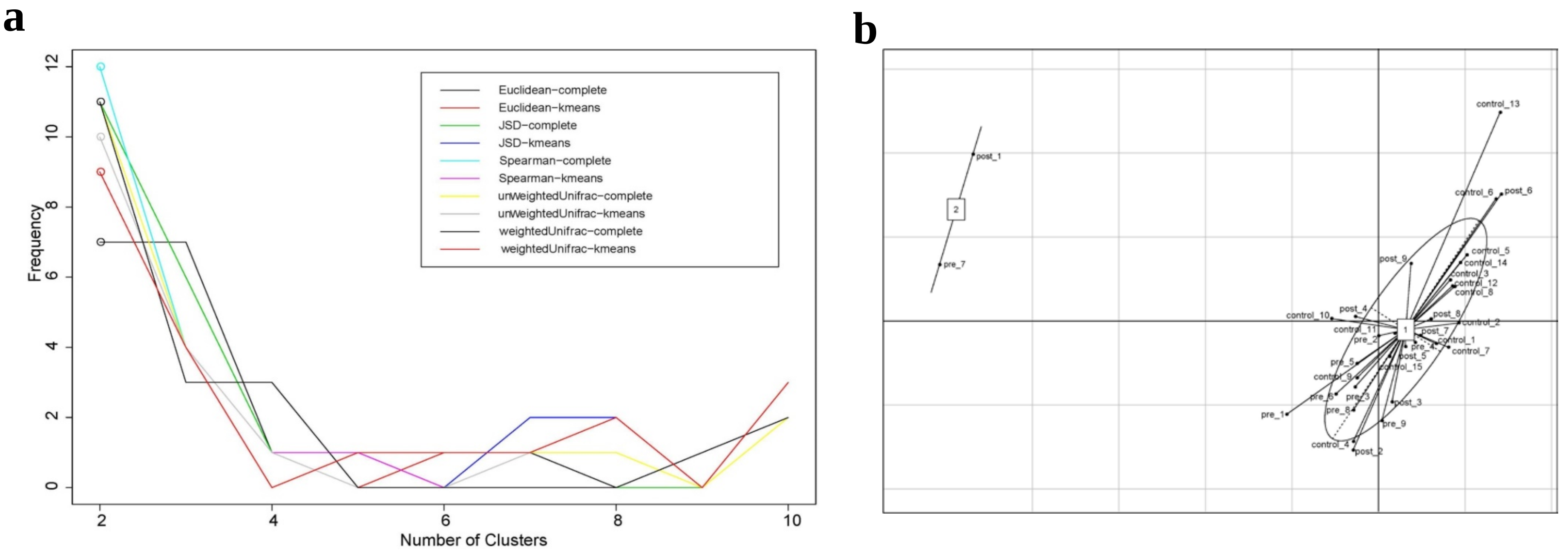

Supplementary Fig.2 a) Cluster frequency diagram under different clust indices and distances: the optimistic number of cluster was 2 according a major-rule;   b) PcoA analyis of all samples based on weighted unifrac distance and K-means clusting method, pre-7 and post-1 were binned into a discrete cluster then identified as outliners.

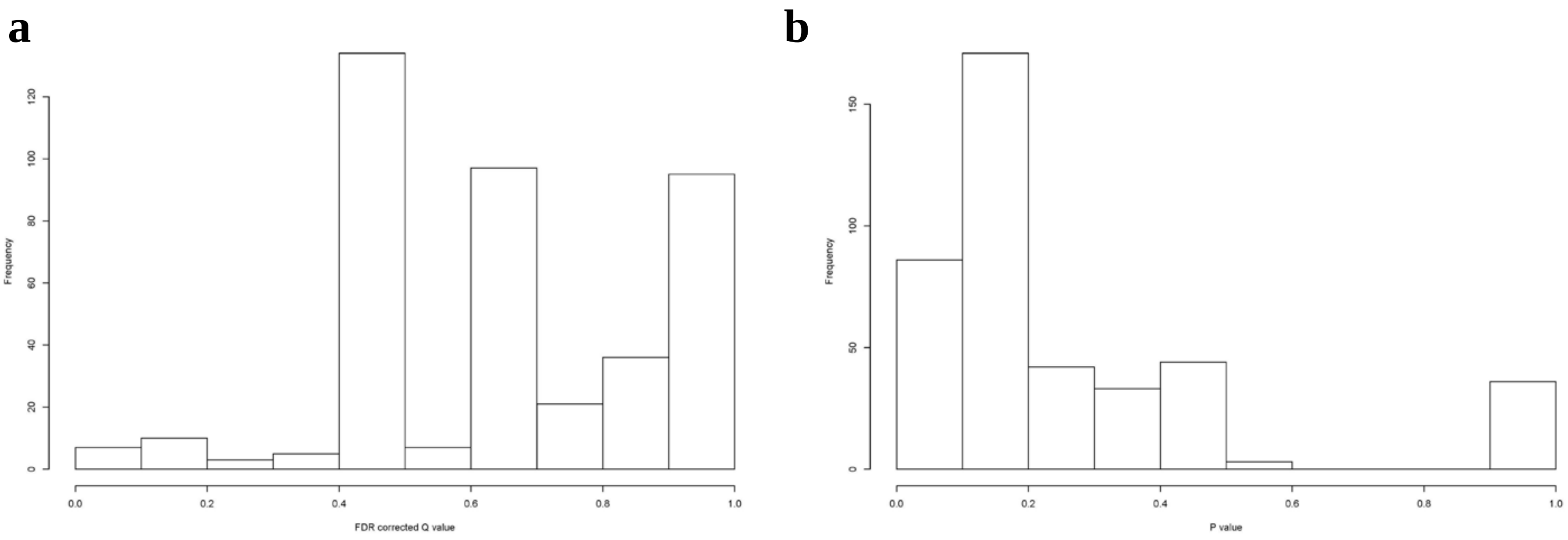

Supplementary Fig.3 Histogram of Pvalues calculated by hypothesis tests based on 7 paired samples. a) FDR corrected q values by multiple testing. b) treated-naive P values.

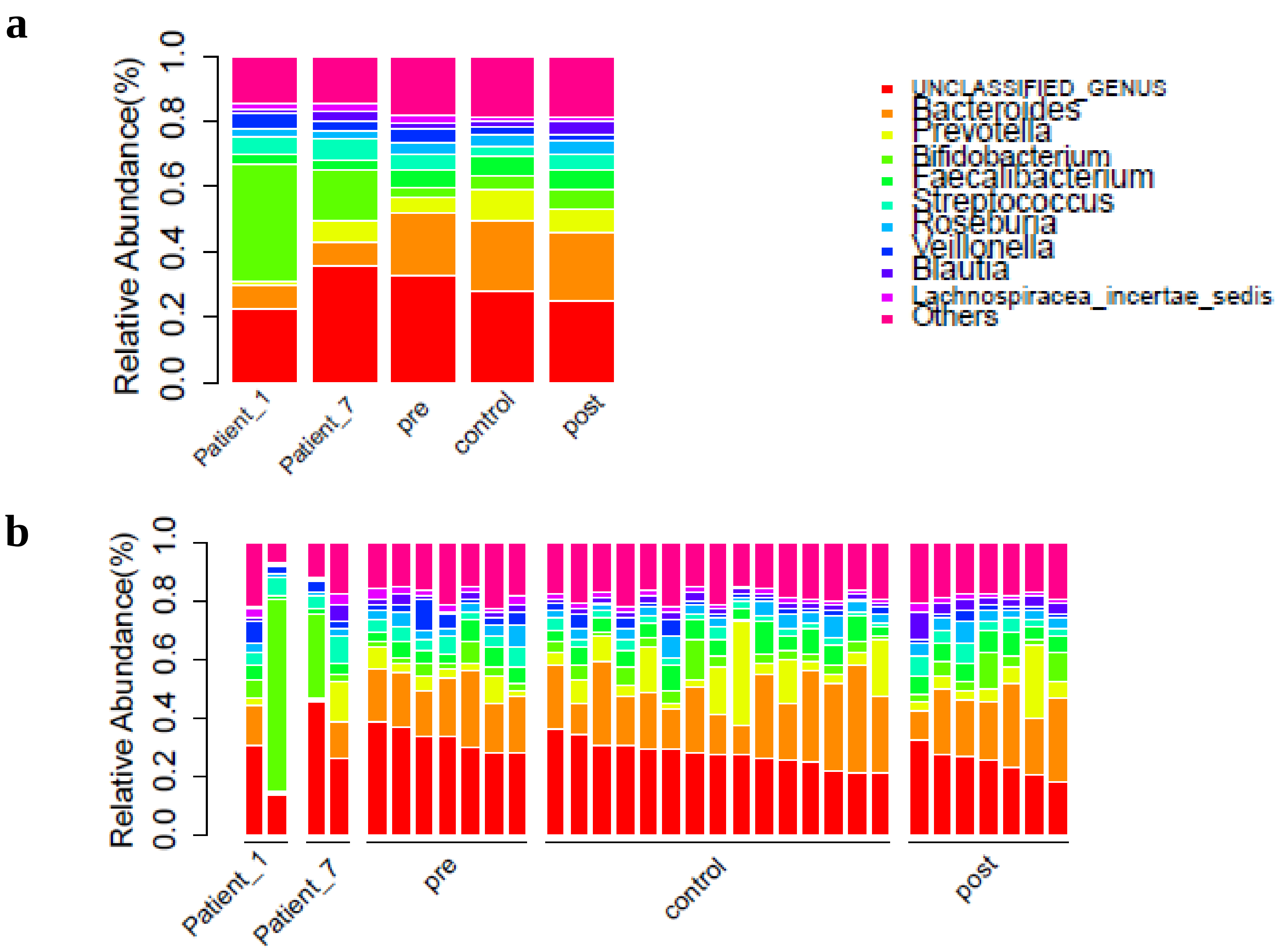

Supplementary Fig.4 Barplot of all microbiota at genus level . a) Mean relative abundance of all cohort. b) Relative abundance of all samples.

Table S1 Characteristics of Patients in Study

Case	Gender	Age	BMI	Diagnosis	MELD
1	male	54	23.4	HBV-related cirrhosis,HCC	23
2	male	53	22.1	HBV-related cirrhosis,HCC	28
3	male	52.3	26.8	HBV-related cirrhosis,HCC	19
4	male	51	21.5	Decompensated alcoholic cirrhosis	16
5	male	64	26.3	Decompensated alcoholic cirrhosis	22
6	male	18	17.6	Decompensated cirrhosis,Portal vein cavernous transformation	18
7	male	65.3	22.7	HBV-related cirrhosis	16
8	male	40	25.5	HBV-related cirrhosis,HCC	12
9	male	48	24.8	HBV-related cirrhosis,HCC	10

Table S2 Clinical data of study population before LT

	Cirrhosis complications of pre-LT				LT indication	Past medical history
	Refractory ascites	Gastrointestinal bleeding	hepatic encephalopathy	malignant tumor		
1	No	No	No	Yes	liver cirrhosis and HCC	No
2	No	No	No	Yes	liver cirrhosis and HCC	TACE
3	Yes	No	Yes	Yes	liver cirrhosis and HCC	TACE
4	No	No	No	Yes	liver cirrhosis and HCC	No
5	Yes	No	No	No	Decompensated liver cirrhosis	No
6	No	No	No	No	Decompensated liver cirrhosis	No
7	Yes	Yes	No	No	Decompensated liver cirrhosis	No
8	No	Yes	No	No	Decompensated liver cirrhosis	Cholecystectomy
9	No	No	No	Yes	liver cirrhosis and HCC	TACE

Table S3 Peri-operative complications of study population

	Hypertension	Hyperglycaemia	Biliary complication	Bleeding	Infection
1	No	No	No	No	Pneumonia ，Healing after 10 days
2	No	No	Biliary anastomotic stricture	No	No
3	No	No	No	Yes，the second day post-LT, hemostasis was performed	No
4	No	Yes，use Insulin	Biliary anastomotic stricture	No	No
5	No	No	No	No	No
6	Yes，take Norvasc	No	No	No	No
7	No	No	Slight bile leakage	No	No
8	Yes，take Norvasc	No	No	No	No
9	No	No	No	No	No

Table S4 Characteristics of controls in Study

Control	Gender	Age(year)	Height(cm)	Body weight(Kg)	BMI
control_1	male	49	170	66	22.8
control_2	male	46	176	72	23.2
control_3	male	54	170	71	24.6
control_4	male	48	177	74	23.6
control_5	female	50	161	61	23.5
control_6	male	49	172	71	24.0
control_7	male	47	178	79	24.9
control_8	female	46	163	62	23.3
control_9	male	46	177	74	23.6
control_10	female	44	160	55	21.5
control_11	male	59	180	80	24.7
control_12	male	42	170	68	23.5
control_13	male	44	173	69	23.1
control_14	male	47	170	66	22.8
control_15	female	49	158	59	23.6

Table S5 Comparison of healthy controls with patients in Age & BMI & Sex

	sex(F=female,M=male)	Age(mean/sd)	bmi(mean/sd)
Patient	F=0,M=9	49.5/4.19	23.5/0.85
Control	F=4,M=11	48/14.08	23.41/2.87
Pvalue 1	0.2589	0.1691	1
Pvalue 2	0.2579	0.7611	0.9193

Pvalue1: Fisher's exact test(Sex), wilcoxn rank sum test(Age/BMI)

Pvalue2: Chi square test(Sex), student's t test(Age/BMI)

Table S6

	WeightedUnifrac	UnweightedUnifrac	JSD	Spearman
P value (sex)	0.375	0.487	0.308	0.2
P value (age)	0.161	0.506	0.293	0.13
P value (bmi)	0.071	0.067	0.215	0.224
P value (Cohort)	0.004	0.845	0.019	0.353
Sum of Square(Residual)	0.6324	0.47698	1.21051	0.54268

Table S7 Pyrosequencing Data of Each Sample in Study

Sample	Available_reads	Random_chose_reads	OTU(all)	Shannon_index	OUT	chao1
pre_1	62025	10000	625	4.25	321	403.51
pre_2	55026	10000	591	4.32	304	366.13
pre_3	28377	10000	463	3.94	286	354.12
pre_4	31039	10000	520	4.52	339	407.76
pre_5	57307	10000	575	4.00	291	365.06
pre_6	46524	10000	574	4.20	335	425.01
pre_7	50406	10000	513	2.44	254	408.51
pre_8	39244	10000	529	4.29	307	379.00
pre_9	44660	10000	565	4.46	322	418.49
control_1	79899	10000	583	4.01	289	393.90
control_2	46168	10000	507	4.07	270	338.12
control_3	50422	10000	586	4.40	309	367.68
control_4	56930	10000	563	4.49	315	385.05
control_5	15414	10000	404	4.08	289	367.77
control_6	23069	10000	494	4.15	319	409.05
control_7	15251	10000	415	4.41	313	424.11
control_8	50085	10000	524	4.26	293	369.24
control_9	39353	10000	592	4.55	349	469.13
control_10	46680	10000	588	4.28	327	399.43
control_11	18221	10000	434	4.19	312	392.12
control_12	27939	10000	515	4.39	322	388.22
control_13	19515	10000	421	3.56	291	356.19
control_14	40539	10000	564	4.23	311	383.12
control_15	32605	10000	533	4.41	330	380.00
post_1	45019	10000	470	1.82	241	358.56
post_2	29348	10000	526	4.47	324	389.19
post_3	28375	10000	532	4.45	326	414.47
post_4	18782	10000	453	4.31	318	396.05
post_5	34056	10000	579	4.47	326	400.42

**post_6**	21013	10000	465	4.12	317	421.02
**post_7**	36011	10000	533	4.33	318	412.56
**post_8**	54353	10000	574	4.30	323	426.51
**post_9**	37984	10000	505	4.25	312	375.22

Table S8

	pre vs control			control vs post		
	**PC1**	**PC2**	**PC3**	**PC1**	**PC2**	**PC3**
**JSD**	0.1333	0.1842	0.1661	0.4726	0.0193	0.3398
**Hellinger**	0.105	0.1842	0.1842	0.4453	0.0193	0.3149
**Spearman**	0.0193	0.3654	0.1333	0.0926	0.0069	0.0033
**weighted Unifrac**	0.0213	0.0465	0.4902	0.2982	0.8907	0.2101
**unweighted unifrac**	0.2101	0.5815	0.6796	0.2372	0.0038	0.9452
